# Supplementary material for: CtGH76, a Glycoside Hydrolase 76 from Chaetomium thermophilum, with Elongated Glycan-Binding Canyon
Source: Int J Mol Sci. 2025 Jul 9;26(14):6589. doi: 10.3390/ijms26146589 (PMC12295086; doi:10.3390/ijms26146589)
Supplement: Supplementary file 1 [file ijms-26-06589-s001.zip › ijms-3701152-supplementary.pdf]

Supplementary information

*Ct*GH76, a Glycoside Hydrolase 76 from *Chaetomium thermophilum*, with Elongated Glycan-Binding Canyon

Silvana Ruth Ruppenthal †, Wang Po-Hsun †, Mohamed Watad, Christian Joshua Rosner, Marian Samuel Vogt, Markus Friedrich, Anna-Lisa Voigt, Angelique Petz, Petra Gnau and Lars-Oliver Essen *

Faculty of Chemistry, Department of Biochemistry, Philipps-University, 35043 Marburg, Germany; silvana.ruppenthal@chemie.uni-marburg.de (S.R.R.); wangpo@staff.uni-marburg.de (W.P.-H.); watadm@staff.uni-marburg.de (M.W.); christian.rosner@chemie.uni-marburg.de (C.J.R.); marian_vogt@yahoo.de (M.S.V.); friedrich-regensburg@web.de (M.F.); anna-lisa.voigt@pharmazie.uni-marburg.de (A.-L.V.); angelique.petz@gmail.com (A.P.); gnaup@staff.uni-marburg.de (P.G.)

* Correspondence: essen@chemie.uni-marburg.de; Tel.: +49-(06421)-282-2032

† These authors contributed equally to this work.

This PDF file includes:

Supplementary Information: Figures S1 to S8

Supplementary Information: Tables S1 to S3

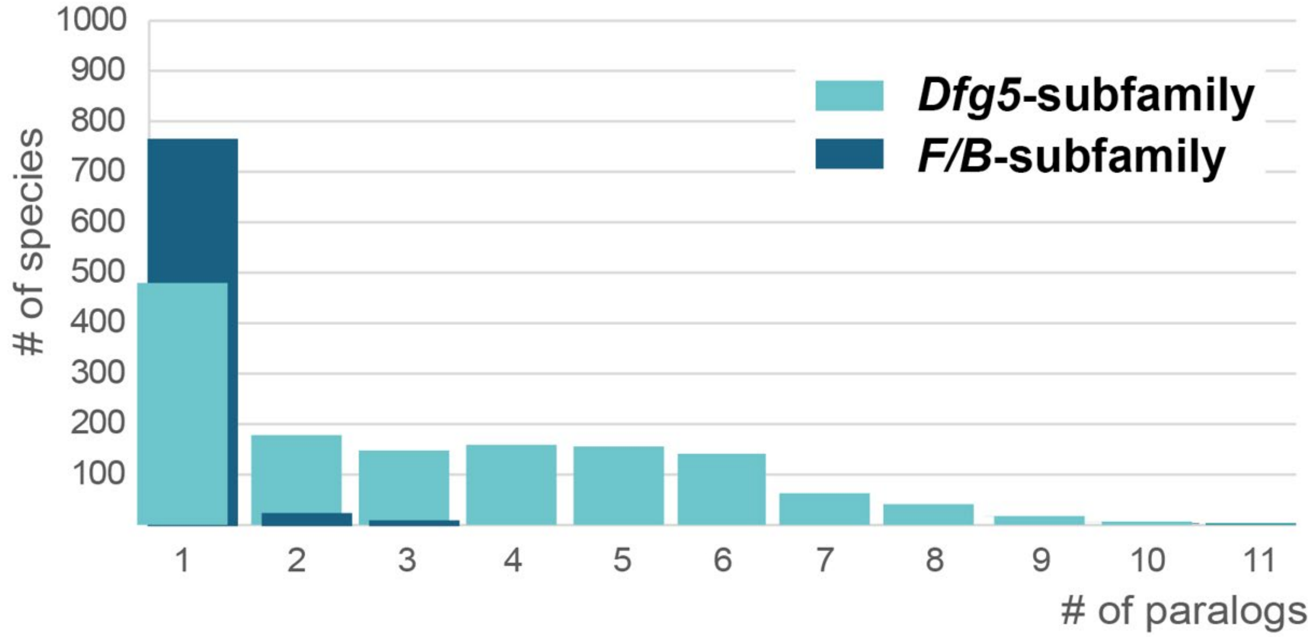

Figure S1. Analysis of the frequency of GH76 paralogs in *Ascomycota*. Paralogs of the *Dfg5*-subfamily (*F*/*B*-mixed subfamily) are found in 1395 (810) ascomycete species. On average 4.1±2.5 paralogs are found in species with *Dfg5* orthologs (median: 4), but only 1.5±0.8 paralogs (median: 1) for species with *F*/*B*-mixed members.

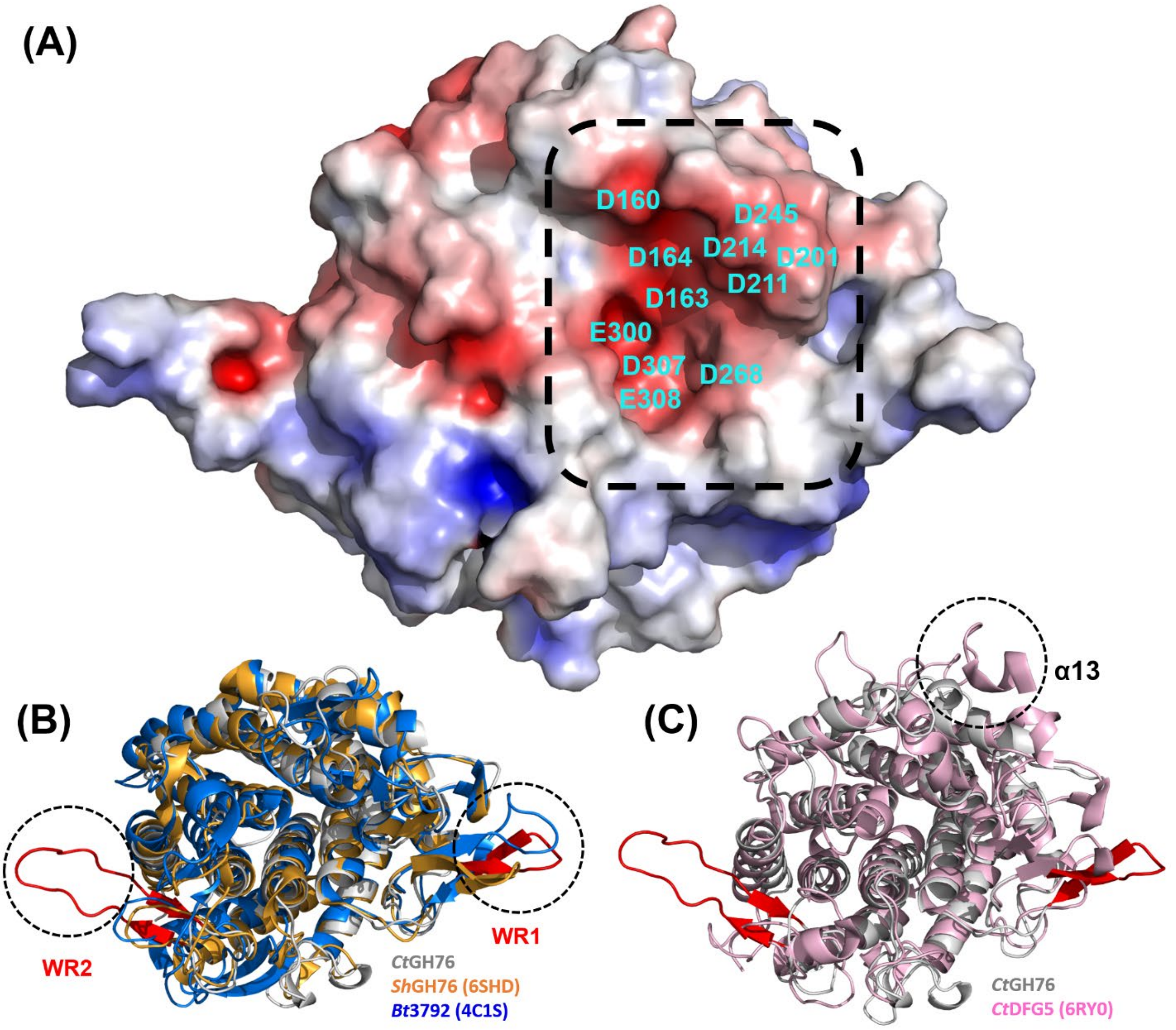

Figure S2. The electrostatic surface potential analysis of *Ct*GH76 and structural superimposition of GH76 members. (A) The electrostatic surface potential analysis was done by APBS and showed the predominantly negatively charged active site in black dashed-box (red: negatively charged; blue: positively charged). Notably, the negative residues within active site are labeled in cyan. (B) Structural superimposition are shown as cartoon models. The *Ct*GH76 was colored in grey, *Sh*GH76 (PDB entry: 6SHD, light orange), *Bt*3792 (PDB entry: 4C1S, blue) and (C) *Ct*Dfg5 (PDB entry: 6RY0, pink). Notably. The extended β-regions of *Ct*GH76 were colored in red, highlighted in the dashed-circle. The additional short α-helix (α13) was highlighted in the dashed-circle in (C).

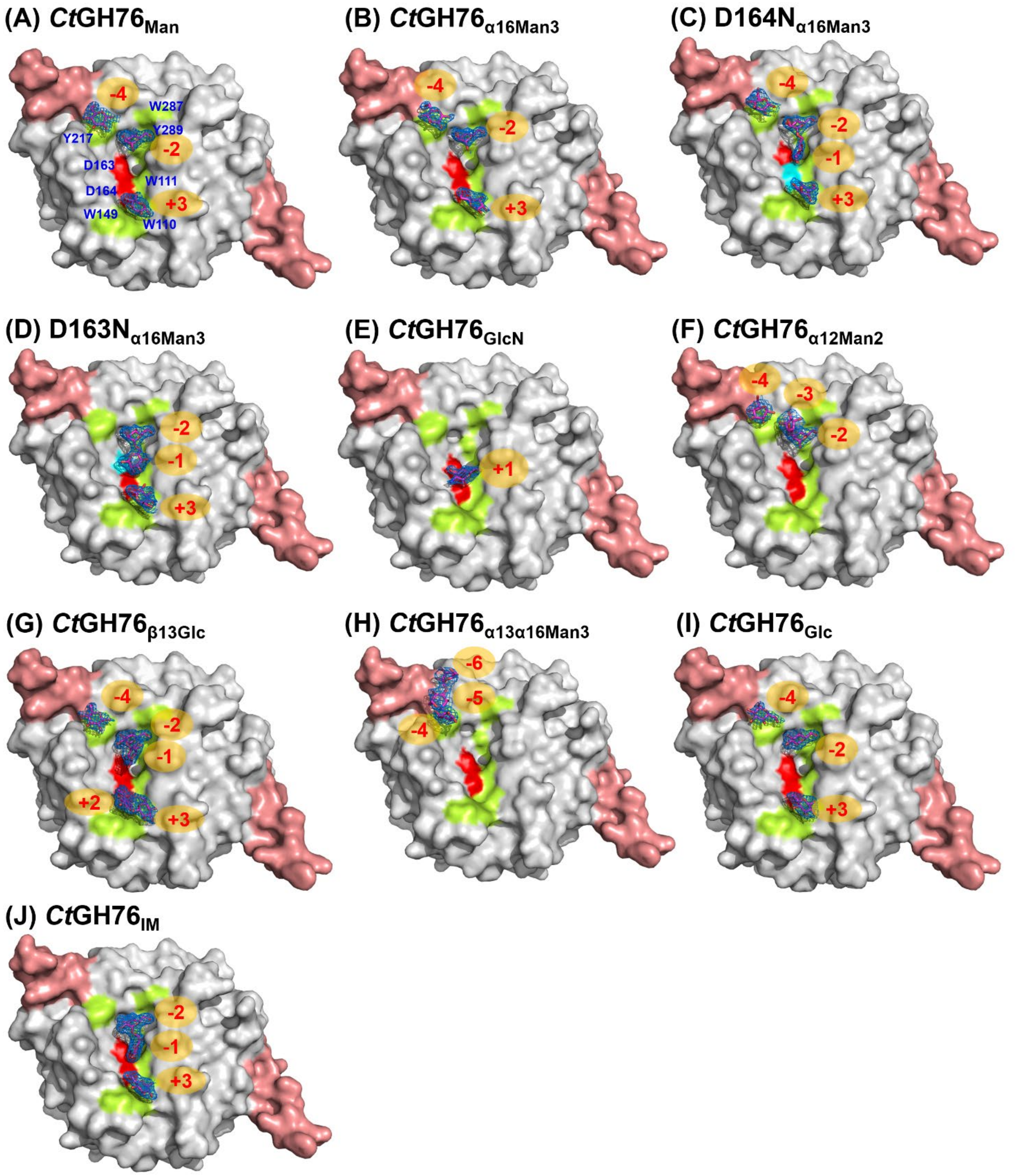

Figure S3. Glycan fragments and omit maps for each structure. The overall protein structures are depicted as grey surface models, with the wing region highlighted in pink. Bound sugar ligands are represented as stick models, accompanied by SigmaA-weighted $2mF_o–DF_c$ electron density maps contoured at the 1.0 σ level and labeled with subsite numbers. Residues interacting with the ligands are marked in green with corresponding residue numbers in (A). The active sites of D163 and D164 are indicated in red, while the mutation is shown in cyan.

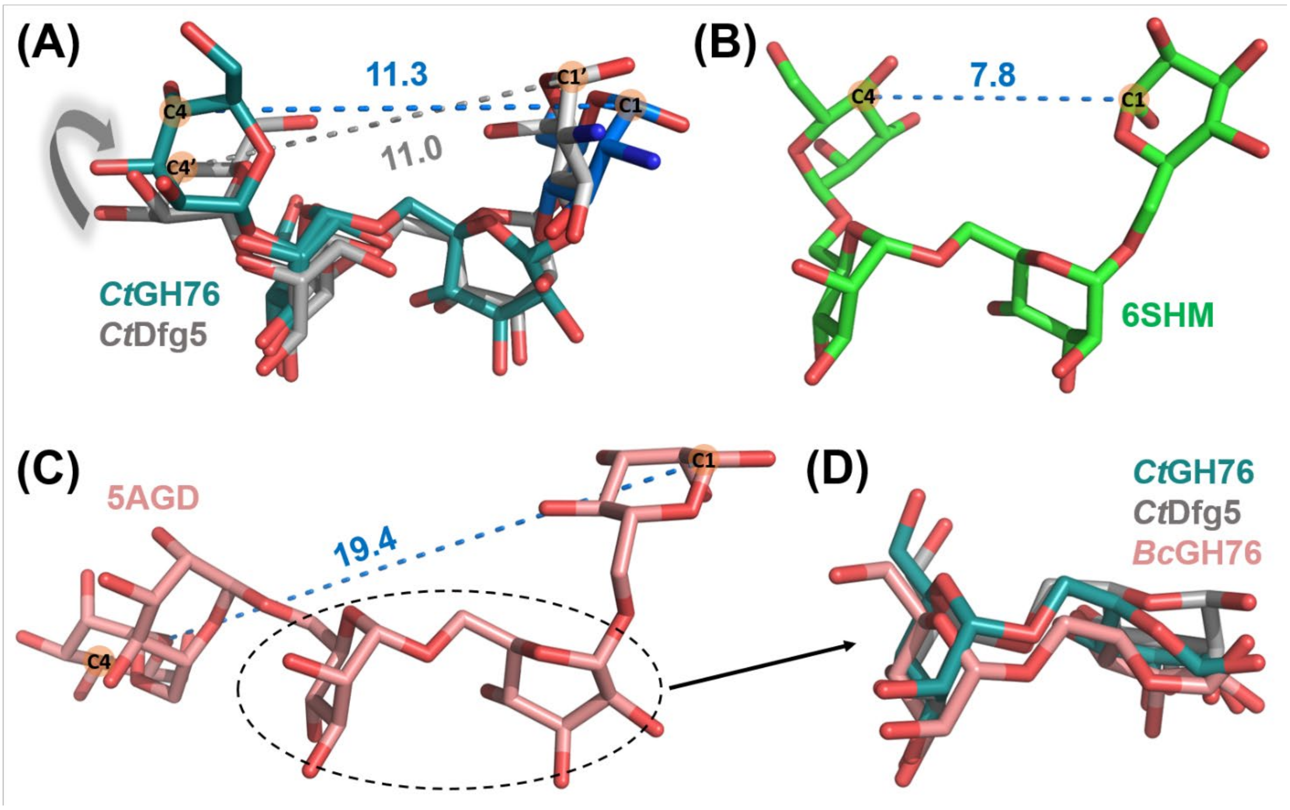

Figure S4. Comparison of GPI anchor and ligand substrates. (A) High structural similarity in the distance between the C1 atom of glucosamine and the C4 atom of the third mannose in the GPI-core glycan. The GPI-core glycan is shown for *Ct*GH76 (blue to dark green, assembled from PDB entries 9R4N, 9R4P and 9R4R), and *Ct*Dfg5 (grey, aligned from PDB entries 6RY2, 6RY5, and 6RY6). (B) Distances between the C1 atom of the first mannose and the C4 atom of the last mannose in α-1,6-linked mannooligosaccharides, depicted for the *Sh*GH76 (green, bound to α-1,6-mannotetraose) and (C) inactive mutant of *Bc*GH76 (pink, bound to α-1,6-mannopentaose). (D) Superposition of subsites -1/-2 in *Bc*GH76, *Ct*Dfg5, and *Ct*GH76. The α-1,6-mannobioses are colored consistently with panels (A) and (C).

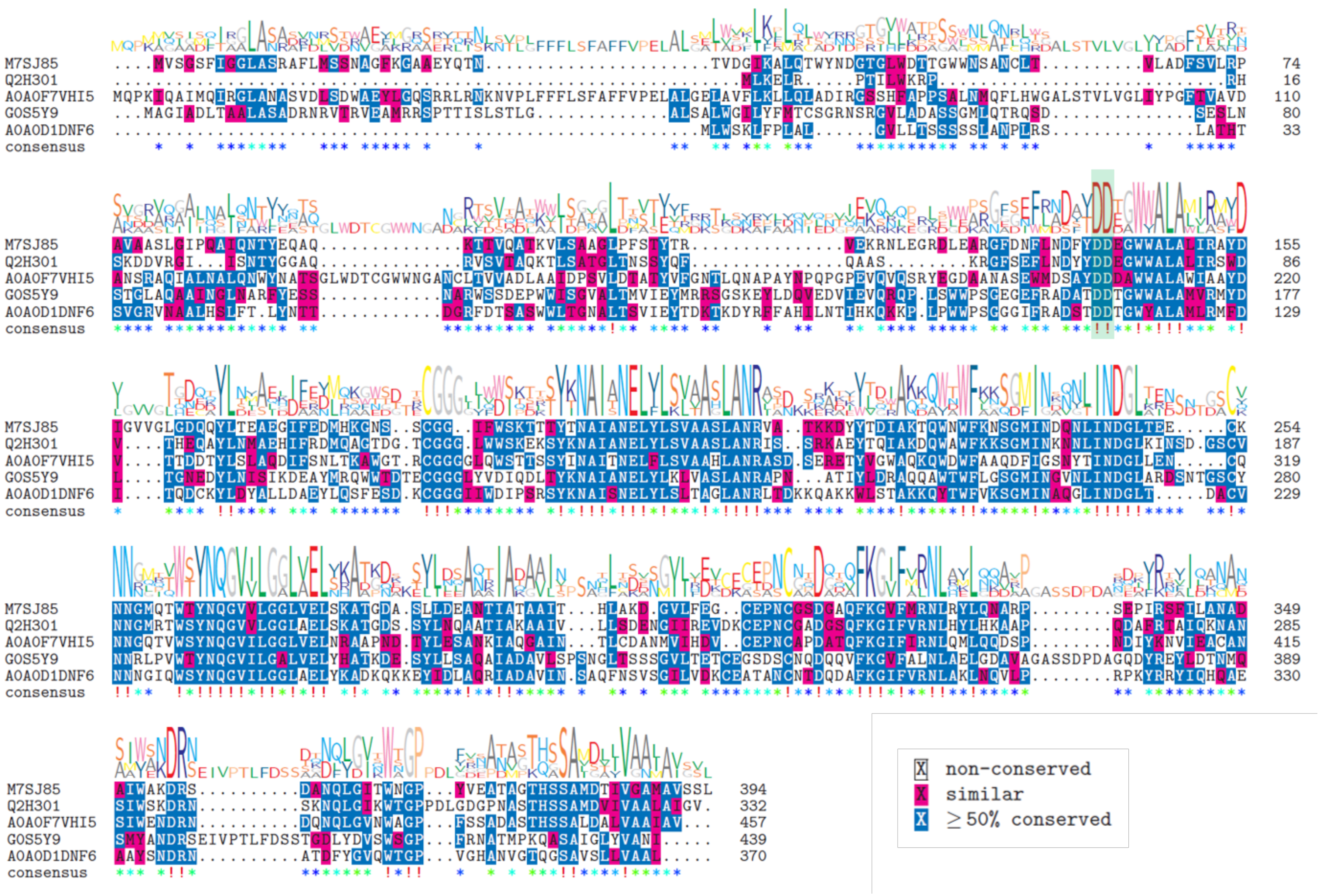

Figure S5. Multiple sequence alignment of members of the *F/B*-mixed subfamily. The protein sequences are aligned using *Chaetomium thermophilum* (Uniprot: G0S5Y9), *Eutypa lata* (UniProt: M7SJ85), *Chaetomium globosum* (UniProt: Q2H301), *Ustilago maydis* (UniProt: A0A0D1DNF6) and *Penicillium brasilianum* (UniProt: A0A0F7VHI5). The conserved DD motif within active site is highlighted in green.

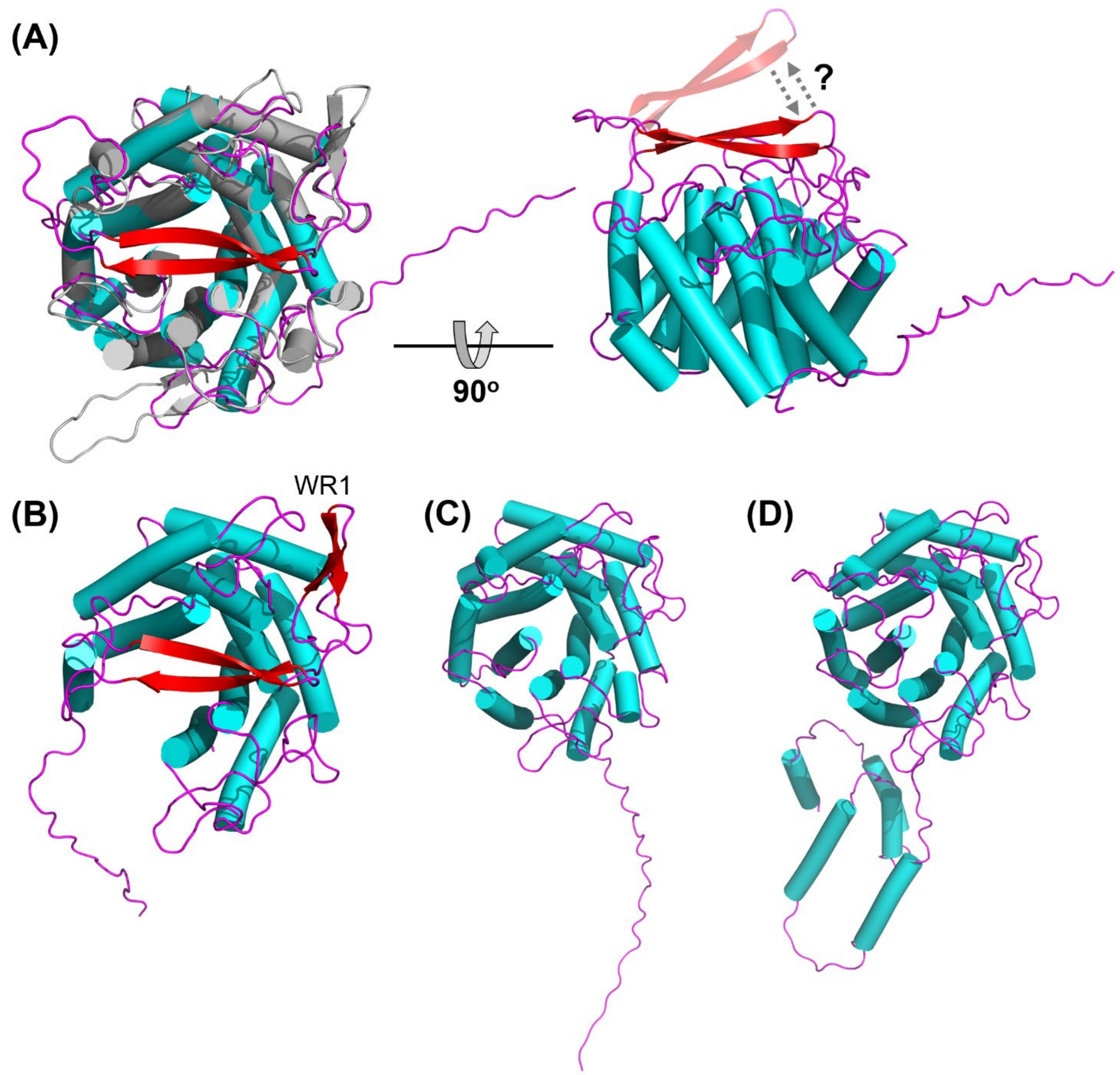

Figure S6. Structural comparison of protein models of the *F*/*B*-mixed subfamily. The fungal models were generated using AlphaFold3 for (A) *Eutypa lata* (UniProt: M7SJ85), (B) *Chaetomium globosum* (UniProt: Q2H301), (C) *Ustilago maydis* (UniProt: A0A0D1DNF6), and (D) *Penicillium brasilianum* (UniProt: A0A0F7VHI5), and are shown in cartoon representation. *Ct*GH76 is highlighted in gray for reference. α-Helices are shown as cyan cylinders, β-sheets as red arrows, and connecting loops in magenta.

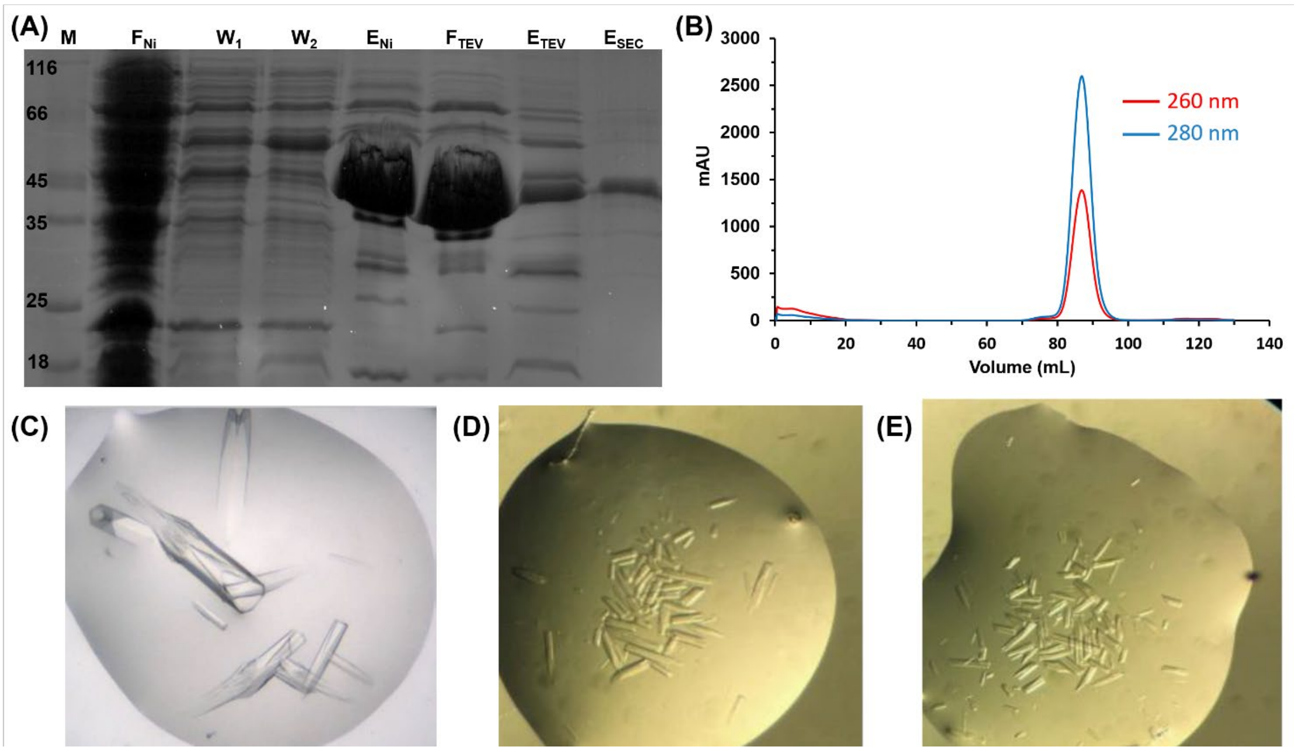

Figure S7. Purification of size exclusion chromatography (SEC) and crystallization of *Ct*GH76. (A) 12% SDS-PAGE gel of *Ct*GH76; F_{Ni}: flow through of nickel-NTA immobilized metal affinity chromatography (IMAC); W_1: wash fraction 1 of IMAC; W_2: wash fraction 2 of IMAC; E_{Ni}: elution fraction of IMAC; F_{TEV}: flow through of IMAC after TEV cleavage; E_{TEV}: elution fraction of IMAC after TEV cleavage; E_{SEC}: elution fraction of SEC; M: protein marker. The purity of *Ct*GH76 was over 95% after final SEC. (B) The SEC chromatogram showed a single major absorbance peak corresponding to the *Ct*GH76 band (calculated mass: ~45 kDa) in the SDS-PAGE. (C) Crystals of *Ct*GH76 wild type, (D) D163N and (E) D164N mutant crystals for synchrotron data collection.

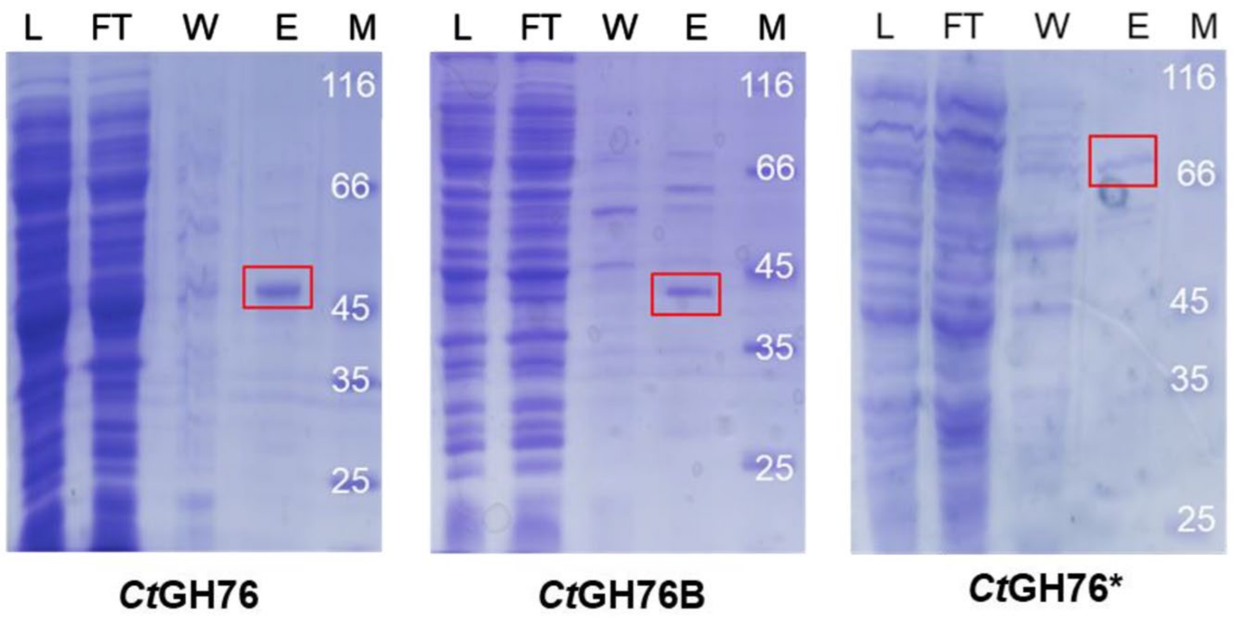

Figure S8. SDS-PAGE of *Ct*GH76 (left), *Ct*GH76B (middle) and *Ct*GH76* (right) following IMAC purification. From left to the right, each gel includes samples for loading (L), flow-through (FT), wash (W), elution (E) and molecular weight marker (M) [kDa]. Notably, *Ct*GH76 displayed the highest yield, ~30 mg/L, and purity among these orthologs.

Table S1. List of glycan fragment soaking experiments for *Ct*GH76.

Structure	Mutation	Ligand	Soaking time
***Ct*GH76$_{Apo}$**	--	--	--
***Ct*GH76$_{Man}$**	--	mannose	180 min
***Ct*GH76$_{\alpha16Man3}$**	--	α1,6-mannotriose	180 min
***Ct*GH76$_{\alpha12Man2}$**	--	α-1.2-mannobiose	180 min
**D163N$_{\alpha16Man3}$**	D163N	α1,6-mannotriose	70 min
**D164N$_{\alpha16Man3}$**	D164N	α1,6-mannotriose	70 min
***Ct*GH76$_{\alpha13\alpha16Man3}$**	--	α1,3-α1,6-mannotriose	180 min
***Ct*GH76$_{\beta13Glc}$**	--	β1,3-glucobiose	180 min
***Ct*GH76$_{GlcN}$**	--	glucosamine	60 min
***Ct*GH76$_{Glc}$**	--	glucose	180 min
***Ct*GH76$_{IM}$**	--	α1,6-glucobiose	120 min

Table S2. Synchrotron data statistics for sugar soaking structures. Numbers in parenthesis describe the highest resolution shell.

Structure	*Ct*GH76$_{Apo}$	*Ct*GH76$_{Man}$	*Ct*GH76$_{\alpha16Man3}$	*Ct*GH76$_{\alpha12Man2}$	D163N$_{\alpha16Man3}$	D164N$_{\alpha16Man3}$	*Ct*GH76$_{\alpha13\alpha16Man3}$	*Ct*GH76$_{GlcN}$
PDB code	9R4K	9R4L	9R4M	9R4N	9R4O	9R4P	9R4Q	9R4R
Ligand soaked	--	mannose	α1,6-mannotriose	α1,2-mannobiose	α1,6-mannotriose	α1,6-mannotriose	α1,3-α1,6-mannotriose	glucosamine
Ligand in structure	--	mannose	mannose	α1,2-mannobiose	α1,6-mannobiose	α1,6-mannobiose	α1,3-α1,6-mannotriose	glucosamine
Mutation	--	--	--	--	D163N	D164N	--	--
Beamline	X06DA, SLS	X06DA, SLS	X06DA, SLS	ID23-1, ESRF	X06DA, SLS	X06DA, SLS	ID30A-3, ESRF	ID30A-3, ESRF
Wavelength (Å)	0.999	0.972	1.000	0.999	0.999	0.999	0.968	0.968
Space group	*P*$3_1$21							
Unit cell (*a*, *b*, *c*)*	106.8, 106.8, 126.9	106.7, 106.7, 127.3	106.5, 106.5, 127.1	107.5, 107.5, 127.2	107.0, 107.0, 127.5	107.1, 107.1, 127.3	107.1, 107.1, 126.5	108.1, 108.1, 128.6
Processing statistics								
Completeness (%)	99.35 (98.14)	99.55 (98.89)	99.18 (96.74)	99.64 (99.68)	99.08 (98.25)	99.31 (98.06)	99.52 (99.44)	97.86 (81.79)
Total reflections	1105758 (101322)	544280 (54932)	1210055 (104475)	300561 (29853)	479943 (46955)	621568 (59762)	481136 (49504)	375167 (38040)
Unique reflections	55077 (5395)	49336 (4860)	60778 (5872)	28187 (2790)	47582 (4668)	31096 (3074)	23534 (2316)	36700 (3630)
Multiplicity	20.1 (18.8)	11.0 (11.3)	19.9 (17.5)	10.6 (10.7)	10.1 (10.1)	20.0 (19.4)	20.4 (21.4)	10.2 (10.5)
CC1/2	1 (0.959)	0.998 (0.967)	1 (0.938)	0.998 (0.955)	0.999 (0.867)	0.998 (0.593)	0.998 (0.872)	0.998 (0.585)
I/σ	31.20 (2.61)	13.57 (2.23)	31.30 (1.94)	7.01 (2.14)	15.46 (1.29)	12.82 (1.16)	15.45 (1.48)	8.73 (0.36)
R_{merge}	0.0149 (0.2827)	0.097 (0.805)	0.051 (1.615)	0.0367 (0.1820)	0.0174 (0.5351)	0.0301 (0.7503)	0.0349 (0.4591)	0.0558 (2.2810)
Wilson B-factor	39.73	42.52	46.84	56.26	53.41	59.10	60.83	60.88
Refinement statistics								
Resolution range (Å)	46.24 - 2.02 (2.09 - 2.02)	46.19 - 2.10 (2.18 - 2.10)	43.36 - 1.95 (2.02 - 1.95)	46.56 - 2.55 (2.64 - 2.55)	46.34 - 2.13 (2.21 - 2.13)	46.37 - 2.46 (2.55 - 2.46)	42.18 - 2.70 2.80 - 2.70	46.82 - 2.35 (2.43 - 2.35)
Unique reflections	54792 (5334)	49145 (4819)	60360 (5810)	28133 (2783)	47291 (4615)	30958 (3034)	23439 (2307)	35956 (2969)
R-work	0.193 (0.368)	0.159 (0.232)	0.168 (0.353)	0.206 (0.315)	0.207 (0.467)	0.202 (0.412)	0.201 (0.415)	0.202 (0.471)
R-free	0.215 (0.364)	0.192 (0.269)	0.193 (0.352)	0.234 (0.322)	0.226 (0.511)	0.221 (0.436)	0.234 (0.533)	0.226 (0.466)
Ramachandran outliers (%)	0.28	0	0.28	0	0.28	0.28	0.28	0.28
Number of non-H atoms	3062	3090	3055	2896	2925	2895	2923	2975
RMS (bonds, Å)	0.002	0.015	0.019	0.002	0.002	0.006	0.005	0.002
RMS (angles, deg)	0.46	1.12	1.54	0.47	0.50	0.72	0.67	0.48
Average *B* factor	57.69	56.77	64.64	71.52	72.6	80.13	69.96	73.01

*α=β=90°, γ=120°

Table S3. Synchrotron data statistics for sugar soaking structures. Numbers in parenthesis describe the highest resolution shell.

Structure	*Ct*GH76$_{\beta13Glc}$	*Ct*GH76$_{Glc}$	*Ct*GH76$_{IM}$
PDB code	9R4S	9R4T	9R4U
Ligand soaked	β1,3-glucobiose	glucose	α1,6-glucobiose
Ligand in structure	β1,3-glucobiose	glucose	α1,6-glucobiose
Mutation	--	--	--
Beamline	X06DA, SLS	ID23-1, ESRF	X06DA, SLS
Wavelength (Å)	0.999	0.999	1.000
Space group	$P3_121$		
Unit cell (*a*, *b*, *c*)*	107.1, 107.1, 126.3	107.2, 107.2, 127.0	107.0, 107.0, 126.8
Processing statistics			
Completeness (%)	99.43 (99.50)	99.60 (99.29)	99.36 (99.03)
Total reflections	456512 (44467)	405792 (40196)	614991 (60499)
Unique reflections	42862 (4235)	38014 (3773)	60612 (5990)
Multiplicity	10.6 (10.5)	10.7 (10.6)	10.2 (10.1)
CC1/2	0.999 (0.929)	0.998 (0.938)	1 (0.944)
I/σ	10.50 (2.17)	11.63 (1.56)	27.05 (1.74)
R_{merge}	0.0229 (0.2762)	0.1065 (0.8632)	0.0103 (0.3166)
Wilson B-factor	48.89	48.85	44.46
Refinement statistics			
Resolution range (Å)	43.52 -2.20 (2.28 - 2.20)	49.40 - 2.30 (2.38 - 2.30)	46.32 - 1.96 (2.03 - 1.96)
Unique reflections	42740 (4217)	37894 (3755)	60294 (5943)
R-work	0.183 (0.296)	0.189 (0.306)	0.179 (0.411)
R-free	0.206 (0.361)	0.209 (0.340)	0.206 (0.440)
Ramachandran outliers (%)	0.28	0.28	0.28
Number of non-H atoms	2953	2968	3019
RMS (bonds, Å)	0.005	0.002	0.019
RMS (angles, deg)	0.69	0.50	1.56
Average *B* factor	66.83	67.36	63.93

*α=β=90°, γ=120°
